# Recruitment and Retention Framework for a Timed-Activity Intervention Among Older Latinos With ADRD

**DOI:** 10.1093/geroni/igab046.809

**Published:** 2021-12-17

**Authors:** G Adriana Perez

**Affiliations:** University of Pennsylvania School of Nursing, Phildelphia, Pennsylvania, United States

## Abstract

Latino participation in ADRD research is essential to advance cognitive health equity. We present results of an adapted framework to increase recruitment and retention of older Latinos with ADRD and caregivers (CGs) in a timed-activity intervention. Framework factors include 3 structures with strategies informed by a Latino Community Advisory Board. For Characteristics of Study Processes, we included linguistically equivalent data collection procedures/measures, scheduled at times most convenient for participants/CGs. Participants were called weekly for questions/guidance with procedures. Intervention sessions built-in additional time to embed Latino cultural values: familismo, personalismo, confianza and respeto. Study Team Infrastructure, included bilingual/bicultural members/students; and trusted community partners to assist with participant referrals. For Preferences and Beliefs Toward Research, we conducted a series of focus groups to understand beliefs about “memory health” and perceptions of ADRD risks. Strategies yielded effective results. We reached our recruitment goal; started a wait-list of interested participants; had zero (n=0) attrition.

